# Biological and Genetic Mechanisms of COPD, Its Diagnosis, Treatment, and Relationship with Lung Cancer

**DOI:** 10.3390/biomedicines11020448

**Published:** 2023-02-03

**Authors:** Karolina H. Czarnecka-Chrebelska, Debjita Mukherjee, Sofya V. Maryanchik, Magdalena Rudzinska-Radecka

**Affiliations:** 1 Department of Biomedicine and Genetics, Medical University of Lodz, 5 Mazowiecka Str. (A-6 Building), 92-215 Lodz, Poland; 2 Institute of Physical Chemistry, Polish Academy of Sciences, Marcina Kasprzaka 44/52, 01-224 Warszawa, Poland; 3 Endocrinology Research Centre, Dmitriya Ul’yanova 11, 115478 Moscow, Russia; 4 Recumed Ltd., Kolejowa 55, 05-092 Łomianki, Poland

**Keywords:** chronic obstructive pulmonary disease, lung cancer, biomarkers, diagnosis, treatment

## Abstract

Chronic obstructive pulmonary disease (COPD) is one of the most prevalent chronic adult diseases, with significant worldwide morbidity and mortality. Although long-term tobacco smoking is a critical risk factor for this global health problem, its molecular mechanisms remain unclear. Several phenomena are thought to be involved in the evolution of emphysema, including airway inflammation, proteinase/anti-proteinase imbalance, oxidative stress, and genetic/epigenetic modifications. Furthermore, COPD is one main risk for lung cancer (LC), the deadliest form of human tumor; formation and chronic inflammation accompanying COPD can be a potential driver of malignancy maturation (0.8–1.7% of COPD cases develop cancer/per year). Recently, the development of more research based on COPD and lung cancer molecular analysis has provided new light for understanding their pathogenesis, improving the diagnosis and treatments, and elucidating many connections between these diseases. Our review emphasizes the biological factors involved in COPD and lung cancer, the advances in their molecular mechanisms’ research, and the state of the art of diagnosis and treatments. This work combines many biological and genetic elements into a single whole and strongly links COPD with lung tumor features.

## 1. Introduction

Chronic obstructive pulmonary disease (COPD) is characterized by lung airflow limitation and tissue destruction; it is the third leading cause of death worldwide [1]. COPD can result from chronic bronchitis with characteristic airway inflammation and scarring [2]. Tobacco smoking is the most common COPD risk factor. However, many other inhaled irritants are still involved (burning biomass fuels, smoke, air pollutants, and chemicals), leading to heterogeneous COPD phenotypes [3,4].

Next, numerous studies have highlighted the strong relationship of COPD with lung cancer (LC) and have represented COPD as a significant LC risk factor independent of smoking behavior [5,6,7].

In this review, we summarized the current research on the pathogenesis and role of biological factors, such as inflammation, oxidative stress, and protease roles in COPD and LC expansion. In addition, we underlined the genetics of the diseases (gene polymorphisms and epigenetic mechanisms), which can cause COPD/LC appearance and its varied severity. Furthermore, we reviewed the current COPD/LC knowledge about diagnoses and treatments. This work aims to evaluate all the factors which link COPD with LC, point out their genetic connections, and underline the vital need for deeper molecular investigation, which can help accelerate their early diagnosis and more efficient treatment.

## 2. COPD Causes

Chronic obstructive pulmonary disease (COPD) involves the chronic inflammatory condition of the lung, particularly the conducting airways and parenchyma [8]. Imbalances accompany the process of progressive inflammation in proteinase/anti-proteinase activity or oxidant–antioxidant balance, triggering emphysema formation (the abnormal enlargement of the air spaces located peripherally from the terminal bronchioles and destruction of the walls of these structures) [1,2,3]. Emphysema may lead to further changes in lung tissue, i.e., deterioration of elasticity, poor expiratory flow, gas trapping, and impairment of the gas exchange [2].

COPD affects millions worldwide, making it a significant health burden connected with high healthcare costs [9]. COPD is attributed to increasing morbidity and mortality in low- and middle-income countries, especially in acute exacerbation patients [10]. The impact of the COPD issue is that many people are underdiagnosed, and only 50% of patients are adequately treated with medications [11].

**Figure 1:**
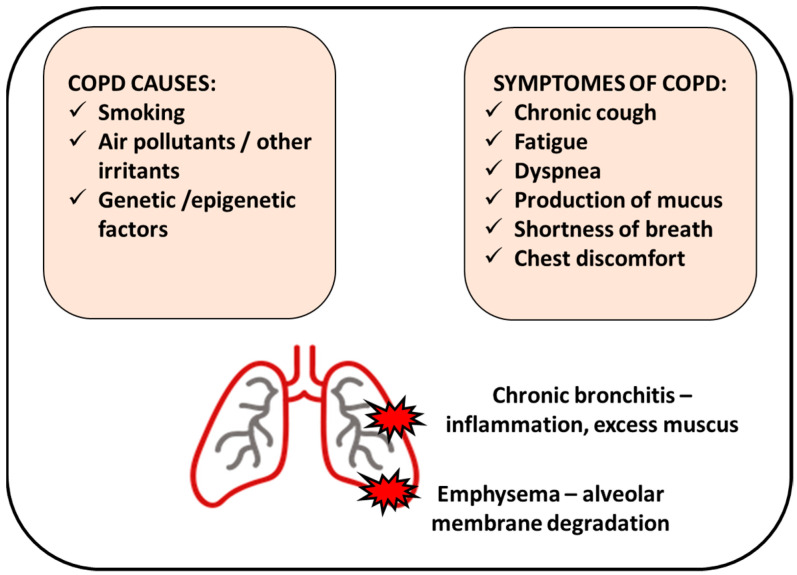
Chronic obstructive pulmonary disease (COPD) manifests by inflamed airways and damaged lung tissue. Smoking cigarettes is the most common cause of COPD; however, other factors can be involved. COPD includes chronic bronchitis (inflammation of the bronchial tubes that causes a persistent cough) and emphysema (damage of the air sacs).

Tobacco smoking is the most common risk factor for developing COPD, with patients more likely to develop the disease if they smoked one pack per day for 20 years or more [3]. However, the absolute risk of COPD developing in continuous smokers is around 25%, suggesting that there may be other predisposing factors, such as genetic, epigenetic, or host-dependent factors (Figure 1).

Among these factors are gene polymorphisms in molecules, such as α1-anti-trypsin [12], tumor necrosis factor (*TNF-α*) [13], matrix metalloproteinases (*MMP*s) [14], or antioxidant gene dependence [15], which show race, age, and disease severity/phenotype dependence [16,17,18].

Next, the disease can be developed from inhalation of smoke from burning biomass fuels, exposure to pollutants and chemicals, or other inhaled irritants [4].

The characteristic COPD symptoms are dyspnea, forced expiratory volume in 1 s (FEV_1_), cough, and sputum production; less common symptoms are wheezing, tightness, and chest congestion [19]. However, reported symptoms showed seasonal and weekly variability and differed depending on the patient population’s disease severity. Frequent exacerbations in COPD patients may eventually result in increased airway inflammation with higher levels of eosinophils and neutrophils and enhanced inflammatory mediators such as cytokines [20].

All cigarette smokers have some lung inflammation, but those who develop COPD present an enhanced or abnormal response to inhaling toxic agents [21]. This amplified response may result in mucous hypersecretion (chronic bronchitis), tissue destruction (emphysema), and disruption of regular repair and defense mechanisms, causing slight minor inflammation and fibrosis (bronchiolitis) [21] (Figure 1).

Two major pathologic processes cause the progressive airflow limitation in COPD: remodeling and narrowing of small airways and destruction of the lung parenchyma with consequent destruction of the alveolar attachments of these airways as a result of emphysema [22] (Figure 1).

After smoking cessation, the symptoms usually decrease. However, lung tissue transformation and lung function are not restored, and increased airway resistance persists. Furthermore, the inflammation persists, contributing to the irreversibility of the decreased lung function. This can be associated with pathological tissue-like fibrosis and inflammation that reduces the diameter of the airway lumen [23].

Due to the overlapping deterioration of processes controlling lung physiology, ageing is regarded as an independent COPD risk factor. The age-related aspects are (i) the decline in the strength of the respiratory muscles due to cardiac function, (ii) age-related reduction in peripheral muscle mass (related to decreased physical activity and nutritional status), and (iii) the geometric changes in the rib cage [24]. Moreover, due to the progressive dilatation of the alveolar ducts and loss of supporting tissues for the peripheral airways, the static elastic recoil of the lung diminishes. Thus, the ageing population is more vulnerable to COPD due to physiological changes in the lung. Since life expectancy has risen, and about 20% of people in developed countries are over 65 years old, focusing on the process leading to lung dysfunction is highly important.

## 3. Genetics of COPD

### 3.1. Gene Polymorphisms

Genome-wide association studies have been conducted to find the genes responsible for the onset or progression of COPD [25] (Table 1). One detected gene directly responsible for COPD appearance is alpha-1-antitrypsin (*AAT*) [26]. AAT deficiency, due to alterations in DNA sequence, is associated with disease development in 1–2% of the affected population. Occurrence of specific alleles, ATT*Z allele homozygosity (Pi Z) or heterozygosity of the Z allele with a null allele, is related with AAT deficiency. In Pi Z homozygotes, the AAT protein polymerize reduces the amount of protein circulating in the body and causes a decreased serum level of AAT [27]. The accumulation of AAT in the hepatocytes leads to liver disorders, such as cirrhosis, hepatitis, and cancer [28]. Since AAT acts as a plasma protease inhibitor of the enzyme leukocyte elastase (present in neutrophils), AAT deficiency leads to loss of the natural defense mechanisms due to lack of proper protease activities and results in inflammation that triggers emphysema, a common condition observed in COPD patients [27,29].

Significant genes connected with susceptibility towards COPD showed a nucleotide polymorphism (SNP) pattern with point mutations causing the replacement of a nucleotide with another in a particular gene locus, resulting in different alleles [30]. In a case-control cohort study conducted by Pillai et al. (2009), two SNPs were observed at the α-nicotinic acetylcholine receptor (*CHRNA3/5* in chromosome 5) locus to be significant in lung dysfunction and increasing the risk for COPD (12.2% cases in the population presented this gene modification) [31]. Extensive cohort investigations of 1633–3000 individuals involving controls (smokers) and COPD patients were studied for SNPs and their pedigree analysis. The two SNPs (rs8034191 and rs1051730) in the *CHRNA3/5* locus were found to be the most reliably associated with COPD and significantly associated with lung function or the FEV_1_ parameter [32]. Furthermore, the observation provided by Wilk et al., 2012, showed that *CHRNA3/5* is a risk factor independent of smoking [33].

Next, the *HHIP* (Hedgehog interacting protein) locus in chromosome 4q31, a part of the hedgehog gene family, was involved in morphogenesis and lung development [31,32,33].

In the same region on chromosome 15q25.1, the *IREB2* (Iron responsive element binding protein 2) and *ADPHD1* (Aspartate beta-hydroxylase domain containing 1) were found to be involved in COPD development. The *HTR4* (5-hydroxytryptamine receptor 4) gene was also responsible for FEV_1_/FVC changes. Another important locus was identified on chromosome 19q13, where the *CYP2A6* (Cytochrome P450 family 2 subfamily A member 6) gene was significant in smoking populations [34,35,36]. The *CYP2A6* gene controls the enzyme required for nicotine metabolism and is vital in smokers. A study by Bloom and colleagues also implicated the gene responsible for hypoxia, *EGLN2* (Egl-9 family hypoxia inducible factor 2), playing a role in COPD; furthermore, *CYP2A6* acts independently of the nicotine metabolism and hence can be responsible for COPD in both smokers and non-smokers [37]. Other genome-wide association studies and meta-analysis examinations have also shown the identification of 39 new loci (such as *EEFSEC*, *DSP*, *MTCL1*, and *SFTPD*) relating COPD to lung function, asthma, pulmonary fibrosis, lung composition (cells, tissues, and smooth muscles) and other comorbidity factors [38,39].

**Table 1:** Gene polymorphisms in COPD.

Gene Identified	Location of Polymorphisms	Critical Effects	Reference
α-1-antitrypsin (*AAT*)	ATT*Z allele (Pi Z) homozygosity, single amino acid substitution causing base pair changes	low levels of AAT in serum, accumulation in hepatocytes leading to liver damage, neutrophil inactivity, emphysema	[26,27,28,29]
Alpha-nicotinic acetylcholine receptor	2 SNPs (rs8034191 and rs1051730) at locus of CHRNA3/5 in chromosome 5	lung dysfunction (deviations in FEV_1_ parameter)	[31,32]
*HHIP* (Hedgehog interacting protein)	chromosome 4q31 (*HHIP* mutations)	developmental problems in the lung and abnormality during morphogenesis	[31,32,33]
*IREB2* (Iron responsive element binding protein 2)	chromosome 15q25.1 (SNP rs7937)	lung developmental changes and emphysema	[34,40]
*ADPHD1* (Aspartate beta-hydroxylase domain containing 1)	chromosome 15q25.1	airflow obstruction, AAT deficiency	[34]
*HTR4* (5-hydroxytryptamine receptor 4)	chromosome 5q31-q33	FEV_1_/FVC changes, airflow obstruction	[36]
*CYP2A6* (Cytochrome P450 family 2 subfamily A member 6)	chromosome 19q13	nicotine metabolism affected	[34,37]
*EGLN2* (Egl-9 family hypoxia inducible factor 2)	chromosome 19q13.2	hypoxia response destroyed	[37]

### 3.2. Epigenetic Regulation (Methylation and Deacetylation)

DNA methylation is a reversible modification of DNA structure involving the transfer of a methyl group onto the C5 position of the cytosine, often as part of a CpG island or cluster [41]. DNA methylation is found to play a critical role in COPD development, and this epigenetic mechanism can be altered by cigarette smoking (Table 2) [42]. Lung macrophages substantially affect the polarization of innate and adaptive immunity and the recognition and elimination of bacteria. In this context, it was detected that several inflammatory/immune-related genes of lung macrophages, including *HSH2D* (Hematopoietic SH2 domain containing), *SNX10* (Sorting nexin 10), *CLIP4* (CAP-Gly domain containing linker protein family member 4), and *TYKZ* are 95 CpG loci with significant difference of methylation [43]. As the authors confirmed, this DNA methylation of selected gene loci in lung macrophages is associated with metabolic differences regionally in the lung.

Next, mitochondrial transcription factor A (mt*TFA*) was remarkably decreased in the skeletal muscle of COPD patients, which was enhanced by cigarette smoke [44]. This phenomenon was positively correlated with the initiation and progression of COPD [44].

Interestingly, the methylation pattern changes can be affected by air pollution components like particulate matter (PM), ozone, nitrogen oxides, and polyaromatic hydrocarbons [45]. Twenty-seven differentially methylated regions (DMRs) in CpGs in *NEGR1* (Neuronal growth regulator 1), *ARID5A* (AT-rich interaction domain 5 A), *FOXl2* (Forkhead box 12), *WDR46* (WD repeat domain 46), *AKNA* (AT-hook transcription factor), and *SYTL2* (Synaptotagmin like 2) genes were correlated with prolonged exposure to PM_10_ and nitrogen dioxide [46].

Differentially methylated regions were detected in parenchymal fibroblasts in COPD, located in genes such as *TMEM44* (Transmembrane protein 44), *RPH3AL* (Rabphilin 3 A like), *WNT3A* (Wnt family member 3 A), *HLA-DP1* (Major histocompatibility complex, class II, DP beta 1), and *HLA-DRB5* (Major histocompatibility complex, class II, DR beta 5) [47]. In addition, GWAS suggested that common *SERPINA1* variants might influence COPD risk and associated lung function phenotypes [48]. Furthermore, hypermethylation of *SERPINA1* in COPD patients was associated with tobacco addiction [49]. As a result, this epigenetic change can affect excessive mucus secretion and production, and goblet cell metaplasia can cause COPD.

Histone acetylation is a reversible epigenetic change unequivocally associated with increasing the propensity for gene transcription [50]. Histone deacetylation and histone acetylation comprise two enzyme families (histone acetyltransferases (HATs) and histone deacetylases (HDACs) [51] and play an influential role in the occurrence of inflammation in COPD [52]. Studies proved the changeability of the acetylation/deacetylation balance toward acetylation in patients with COPD (Table 2) and resultant inflammation [53]. An increase in acetylated histone 4 was found in current smokers; conversely, ex-smokers with COPD showed an increase in histone 3.

Cigarette smoking and oxidative stress are two significant features to inhibit inflammation in lung parenchyma and airways in COPD cases. Consequently, cigarette smoking elevated oxidant stress and promoted COPD glucocorticoid resistance during patients’ treatment. It was associated with higher HDAC2 activity [54]. Ding et al. reported Trichostatin A-an inhibitor of HDAC1/2-suppressing skeletal muscle atrophy and histomorphological alteration in COPD individuals [55]. H3K9 histone acetylation was high in the COPD-diseased human bronchial epithelial group [56].

**Table 2:** Gene methylation and histone acetylation in COPD.

Epigenetic Mechanism	Altering Factors	Targets	Phenotype/Function in COPD Context	Reference
Methylation	cigarette smoking, air pollution	*HSH2D*, *SNX10*, *CLIP4*, *TYKZ*	regulation of lung macrophage activity, maintaining lung metabolic balance	[43]
		mt*TFA*	hypermethylation of the promoter is associated with the initiation and progression of COPD	[44]
		*NEGR1*, *ARID5A*, *FOXl2*, *WDR46*, *AKNA*, *SYTL2*	air pollution-dependent regulation of gene expression in Asians	[46]
		*HLX*, *SPON2*	alteration of functional gene expression in parenchymal fibroblasts	[57]
		*IREB2*, *PSMA4*	smoke-independent association of COPD with genetic variants in chromosome 15q25.1	[58]
Acetylation	cigarette smoking, regulators of HDACs activity (Trichostatin A)	Histones: H3K9, H3, H4	increased levels are associated with inflammation, active gene transcription	[56]

### 3.3. Transcriptional Regulation and Splicing

Transcriptional regulation (effective mechanism in proteostasis) in COPD is affected by malfunction of factors, including β-catenin, TGF-β1, and SMAD signaling. These molecular changes are accompanied by an epithelial to mesenchymal transition (EMT) [59] that can result in organ fibrosis or malignant tumorigenesis [60]. The transcription factor clusters of β-catenin/Snail1/Twist were upregulated, translocated in the nucleus in COPD patients, and correlated with EMT activity and airway obstruction [61]. Next, TGF-β1 was enhanced in COPD samples but was not found to be related to EMT or airflow obstruction. On the other hand, pSMAD was upregulated in the smoking COPD populations and directly associated with EMT and airflow obstruction. mRNA post-transcriptional alterations in COPD cases were closely related to oxidative stress and inflammation [62] and were mediated by factors like RNA-binding proteins (RBPs), microRNA (miRNA), and long non-coding RNA (lncRNA) and posttranscriptional modifications, such as polyadenylation, pre mRNA splicing, and mRNA editing and turnover [63].

Non-encoding RNAs that are not translated into proteins are mainly divided into two categories based on size: (i) short-chain non-coding RNAs (including miRNAs) and (ii) long non-coding RNAs (lncRNAs) [64]. Both short and long non-coding RNA structures were found as potential biomarkers in COPD prognostication and diagnosis. Qu et al. reported that the lncRNA *ENST00000502883.1* was decreased in peripheral blood mononuclear cells in COPD patients. At the same time, Qie et al. found that lncRNAs, including *ENST00000447867* and *NR-026690*, were significantly upregulated in CD4+ T cells of COPD samples [65].

Following, *miR-1236* was found to bind to 3/-UTR of *TLR4* mRNA and pose the risk of ventilator-associated pneumonia in COPD patients. *miR-206* was upregulated in skeletal muscle and plasma of COPD patients and associated with advanced disease [66].

*MiR-206* expression was higher in the lung tissues, and the manifestation of *NOTCH3* and *VEGFA* mRNAs was decreased in the COPD group [67]. *MiR-199a-5p* was repressed by approximately four times and enhanced regulatory T cells in COPD patients compared to healthy smokers [68]. Interestingly, the miRs were differently regulated by tobacco smoking and biomass exposure; *miR-34a* was downregulated in COPD exposed to biomass compared with tobacco smokers [69].

Some of the RBPs, including iron-responsive element binding protein 2 (IREB2), Human antigen R (HuR), and T-cell antigen 1 (TIA-1), are highly expressed in the lungs of COPD patients. In *IREB2* knockout mice, smoke-induced pulmonary inflammation was found, but mucociliary airway clearance was regular [70,71,72,73]. Next, the *miR-218-5p* in human bronchial epithelial cells upon cigarette smoke extract exposure leads to higher overexpression of chemokines, such as *CCL20* and *CXCL8*, which play an essential role in COPD progression [74]. 

The genes associated with COPD progression differ from normal genes due to the transcriptional complexity of the gene *loci* that can produce up to 2.65 transcripts per gene— this is much more than the expected number of transcripts [75]. This phenomenon was connected with these genes’ susceptibility to alternative splicing [76]. Interestingly, the genes mostly associated with COPD, like *SERPINA1* and *AGER*, showed highly regulated expression of splice variants in lung tissue [77,78], and splice variants were replaced with alternative splicing products. This link of COPD-associated genes may be one possible reason behind the development and progression of the disease.

## 4. Pathogenesis of Lung Cancers

### 4.1. Lung Cancer Epidemiology and Subtypes

Lung cancer (LC) is one of the most prevalent cancers, with more than two million new cases yearly, constituting 21.2% of all diagnosed cancers in men, and 8.6% among women, after breast and colorectal malignancies. LC is one of the most common causes of death, responsible for more than a million annually. It has a relatively high fatality rate: the overall mortality ratio to incidence is 0.87. LC is the leading cause of mortality in highly developed countries, and lung cancer mortality accounts for 18–20% of deaths due to cancer yearly [79]. LC in women, as compared with men, occurs at a slightly younger age, and almost half of LC cases in patients under 50 years old occur in women, even in those who have never smoked.

Cancer statistics in Poland (data from 2020) show that around 30,000 new LC cases were diagnosed in 2020, accounting for 14.4% of cancer incidence [80]. LC mortality was 23% of all cancer deaths. In men, it is the predominant type of cancer, accounting for 17.8% of the total cancer incidence. At the same time, it is the second most common cancer incidence in women, accounting for 11% of the total cancer incidence right after breast cancer [80,81].

Five-year survival after lung cancer surgery is approximately 10–70% depending on the stage of the tumor. The best prognosis is for early-stage lung cancers (American Joint Committee on Cancer; AJCC stage I)—the 5-year survival rate exceeds 80%. However, only 16% of LC patients are diagnosed at an early stage [82,83]. Unfortunately, most NSCLC patients are diagnosed in an advanced stage; the 5-year survival rate for LCs in an advanced stage (AJCC stages III-IV) is about 6,6% [82,83]. The high mortality of LC cases also results from a high percentage of patients starting their treatment at an advanced stage, once tumors have metastasized. In the case of metastasis, mainly to the brain, the 5-year survival rate of patients drops to only 4% [84].

LC originates in the epithelial cells of the airways. The lesion often starts growing centrally, in the large bronchi, or peripherally (in the periphery of the lung, closer to the chest wall). It can also spread through local infiltration of the anatomical structures of the mediastinum, diaphragm, pleura, and chest wall. Once cancer spreads from the lung, it infiltrates the regional lymph nodes (infiltration LC metastases), most often appearing in lymph nodes located regionally, followed by the second lung, liver, brain, and bone marrow [85].

In clinical classification, depending on the treatment concerns, two main types of LCs are distinguished: (i) non-small cell lung carcinoma (NSCLC), representing almost 80% of all primary LCs, and (ii) small cell lung carcinoma (SCLC), which accounts for approximately 20% of LCs [86]. The SCLC is more insensitive to chemo-and radiation therapies than NSCLC. The NSCLC patients, after cancer resection, may be additionally treated with adjuvant chemotherapy.

Among the NSCLC type, the three main NSCLC subtypes are distinguished: squamous cell carcinoma (SSC; 30–40% of patients), adenocarcinoma (AC; 10–30% of patients), and large cell cancer (LCC; 10% of patients) [87]. SCC carcinoma arises from squamous epithelial cells, morphologically characterized by the proliferation of atypical, often pleomorphic squamous cells [88]. The SCC subtype is more frequent in males and elders and is associated with cigarette smoking. Unfortunately, LC, especially SCC, can be asymptomatic until it reaches a significant size. AC is more common among younger women, Asian populations, or never-smoking patients, and LCC is characterized by rapid growth and distant metastases [89].

### 4.2. Genetics of Lung Cancer

#### 4.2.1. Genetic Susceptibilities to Lung Cancers

Apart from environmental factors, many genetic susceptibilities may be involved in carcinogenesis in the lung, and numerous molecular changes are responsible for different histopathological types of LC. So far, many molecular alterations (including mutations, polymorphisms, and expression changes due to promoter hypermethylation) in many genes localized in different chromosomal regions have been proposed as leading to NSCLC development [90]. Some patients are more vulnerable to this carcinogenic activity due to inherited genetic mutations or polymorphisms (mainly single-cell polymorphisms) impairing the DNA damage repair processes or the inactivation of harmful components of tobacco smoke. For example, polymorphism in the tumor suppressor protein 53 (*TP53*) may weaken or change its function and the Arg72Pro alternative results in a slight structural change accompanied by higher efficiency in cell cycle arrest and DNA repair. This polymorphism increases the LC in heavy smokers (observed for patients with squamous-cell carcinoma); however, the increase in LC depends on the population studied [91,92]. On the other hand, the Arg72Pro variant is related to an elevated risk of developing pancreatic cancer among males, hefty smokers, and excessive alcohol drinkers [93]. Asp353Glu polymorphism in TP53 binding protein 1 (*TP53BP*) can worsen the cellular response to DNA damage and contribute to the increased risk of LC [94]. Another gene whose single nucleotide polymorphism can increase the LC risk is *P21*/*CDKN1A*. The Ser31Arg variant leads to a lower apoptosis rate and extension of G1 cell cycle arrest as a response to the carcinogens (i.e., smoke) [91].

#### 4.2.2. Genetic Factors Involved in the Pathogenesis of NSCLC

LC initiation and progression result from permanent genetic changes that include point mutations, deletions, translocations, amplifications, and epigenetic modifications. Genetic analyses of NSCLC subtypes have shown a higher mutation rate in these tumors compared to acute myeloid leukemia, glioblastoma multiforme, breast cancer, ovaries, and large intestine [95]. The involvement of several genetic factors in the mechanisms of tumorigenesis in the lung has been revealed; however, in about half of the patients, critical molecular change has not been established.

The vast majority of mutant genes encode proteins involved in the receptor tyrosine kinase (RTK) signaling pathway, promoting proliferation, survival, migration, and invasion of NSCLC cells [96]. Some alterations were found in the genes for the tyrosine kinase receptors: *EGFR*, *ALK*, *MET*, *RET*, *FGFR1*, and *DDR2*, or in genes associated with cell signaling mediated by Kirsten rat sarcoma viral oncogene homolog (*KRAS*), B-Raf proto-oncogene, serine/threonine kinase (*BRAF*), and phosphoinositide-3-kinase catalytic subunit alpha isoform (*PIK3CA*) in NSCLC [97]. Another frequently mutated gene is Phosphatase and tensin homolog deleted in chromosome 10 (*PTEN*), having a solid TSG role due to the negative regulation of the PI3K/mTOR/Akt oncogenic signaling pathway. Moreover, the PTEN protein plays a role in chromosomal stability maintenance and activates the RAD51 protein in the DNA repair process [98]. According to the TCGA data, the *PTEN* mutation frequency strongly depends on the LC subtype, occurring in 15–20% of the SCC and only 3% of AC [99]. Early growth response gene 1 (*EGR1*) gene expression predicts *PTEN* levels and survival after surgical resection of NSCLC; lower levels of *EGR1* are associated with poor outcomes [100]. Although the genetic changes occurring in both ADC and SCC affect RTK signaling, these NSCLC subtypes have different dominant mutations, amplifications, or rearrangements [97].

Numerous transcripts in which genetic changes have occurred have been identified in adenocarcinomas, including *EGFR*, *EML4-ALK*, *KRAS*, *MET*, *RET*, *BRAF*, and *TP53* [97,101]. One of the most common genetic changes characteristic of the adenocarcinoma lung cancer subtype is the *KRAS* gene mutation affecting the proliferation and development of chemo-resistance and cancer cell survival [97]. The second most prevalent is the Epidermal growth factor receptor (*EGRF*) gene mutation, occurring in 10–15% of LC patients. *EGFR* mutation increases cancer cells’ proliferation, survival, angiogenesis, and metastasis. There are two therapies targeting the mutated *EGFR*-small-molecule EGFR tyrosine kinase inhibitor (TKI)—gefitinib and erlotinib—and monoclonal anti-EGFR antibody—cetuximab. The L858R missense mutation (exon 21) stands for the vast majority of the *EGFR* kinase mutations (85%) and is sensitizing to EGFR TKIs [102]. Based on the mutational status of the most prevalent genes, *EGFR*, *KRAS*, and *TP53*, lung adenocarcinoma classification into seven prognosis-related subtypes can be performed. EGFR-positive patients that underwent lung surgery had better OS than tumors with co-mutations in *KRAS* and *TP53* [101].

*EGFR*, one of the most commonly modified genes in AC, is rarely altered in the SCC subtype [103]. In the SCC subtype, the mutations mainly concern the genes for *FGFR1*, *PIK3CA*, *DDR2*, *MET*, *SOX2*, *PTEN*, and *CDKN2A* [102]. The two most common mutations in the SCC subtype, present in the majority of SCC patients, are the *TP53* and Cyclin-dependent kinase inhibitor 2A (*CDKN2A*) (mutations [104]. Highly prevalent (accounting for up to ¼ of cases) is the fibroblast growth factor receptor 1 (*FGFR1*) amplification. Altered *FGFR1* is associated with increased cancer cell proliferation and survival but also with the development of chemo-resistance rearrangements [97].

The KEAP1 is part of the Keap1/Nrf2 signaling pathway, which is critical in maintaining homeostasis and protecting against various respiratory tract diseases [105]. This pathway has been linked to oxidative stress, tumor progression, and the development of resistance to chemotherapy. The transcription factor *NRF2* regulates the activity of enzymes involved in carcinogen detoxification [106]. Moreover, mutations in the genes encoding the *Keap1* and *Nrf2* proteins are associated with the short survival time of patients diagnosed with LCC [106].

The diagnosis of molecular changes is helpful in the classification of the neoplasm and prognosis of the course of the disease and also supports the selection of the optimal therapy [107]. The conducted molecular research, including the most modern approach—next-generation sequencing (NGS)—allows for developing an ever more comprehensive panel of assessed molecular markers. In turn, detailed diagnostics broaden the possibility of qualifying NSCLC patients for molecularly targeted therapy.

Detection of Echinoderm microtubule-associated protein-like 4-anaplastic lymphoma kinase (*EMLK4*-*ALK*) and ROS Proto-Oncogene 1, Receptor Tyrosine Kinase (*ROS1*) gene rearrangements in NSCLC is required for directing patient care. For screening the ALK-positive patients, two methods: (i) the fluorescence in situ hybridization diagnostic test (FISH) and (ii) immunohistochemistry assays, have been approved by the FDA [108]. In the cases where established gold standard methods might give false-positive results, the NGS approach is reliable for assessing *ALK* and *ROS1* rearrangement [109]. In the case of *ROS1* tyrosine kinase, many fusions with different proteins are detected, e.g., CD74-ROS1, SLC34A2-ROS1, FIG-ROS1, or CCDC6-ROS, among others [110]. Detection of the ROS1 rearrangements can be performed with FISH break-apart probe methodology and targeted real-time PCR (for already-known fusions) and NGS panels (for novel fusion partners) [108].

In addition to somatic mutations predisposing to lung cancer and mutations acquired from exposure to inhaled carcinogens, genetic instability is an essential factor contributing to lung cancerogenesis. The chromosomal instability via structure and/or number aberration has been claimed as a universal biomarker. The structural alteration of the chromosomes may lead to the loss of heterozygosity-LOH (more frequently) and microsatellite instability—MSI [90]. Those changes may occur both within the coding region of the genes or as microdeletion in the non-coding sequences. Several studies in LC revealed the presence of LOHs in different chromosomal regions: 1p, 3p 7q, 9p, 11p, 12q, and 16q. The allele loss occurring within the most studied area—the short arm of the third chromosome—*loci* of many TSGs, represents the “discontinuous LOH pattern.” LOHs were detected in different concentrations in several sites, starting from the short 600-kb 3p21.3 region. Furthermore, minor and less frequent alterations were observed in preneoplastic/preinvasive respiratory epithelium, and the range of loss increased with cancer progression [111]. Two frequently affected regions (FARs): LUCA (lung cancer region in 3p21C) and AP20 (Alu-PCR clone 20 region, 3p21T), are also located within 3p [112]. In the study performed on the bronchial epithelia of smokers (in the pre-cancer stage), the occurrence of genetic instability in 3p21, 9p21, 17p13, and 5q11 was associated with carcinoma in situ incidences [113]. The high LOH/MSI frequency within the TSPs located in 3p-*RARβ*, *FHIT*, and *MLH1* were correlated with tobacco smoking, indicating the single factor boosting the risk of developing COPD and lung cancer [114]. Many studies on LOH/MSI presence in one genomic region showed contradicting information regarding the association of the genetic change with cancer subtype, stage, or smoking. Therefore, instead of analyzing LOH/MSI in one *locus*, one can rely on the indicator that calculates the percentage of chromosomal loci with LOH/MSI concerning all examined *loci*-Fractional allele loss (FAL) [90].

### 4.3. Epigenetic Susceptibilities to Lung Cancers

Different DNA methylation patterns in cancer cells compared to normal tissues have been studied for more than 25 years. The cancer cell can undergo global genome hypomethylation, including demethylation of the promoter regions of oncogenes or the repetitive DNA, which under physiological conditions are heavily methylated [115,116]. The reverse process may take place in the areas that regulate the expression of the tumor suppressor genes (TSGs), leading to changes in transcription and expression [115,116,117].

Demethylation of the retrotransposons (the mobile elements of the genome) leads to their increased transcription and recombination events, though linking global hypomethylation with genomic instability [117,118]. Long interspersed elements (LINEs), covering almost 17% of the human genome, are observed to be transcriptionally active in many cancers. The LINE-1 region demethylation in LC results in the transposition of the retro-element, increasing the genomic instability and finally, contributing to chromosomal alteration formation. Its hypomethylation in early-stage NSCLC was indicated as a poor prognosis factor. However, *LINE-1* demethylation may suggest that patients need post-surgical adjuvant therapy [119]. On the other hand, *LINE-1* hypermethylation was regarded as a good prognostic factor for LC patients [118].

The aberrant methylation in cancer usually occurs at CpG islands located in promoter regions and the first exon of the TSGs, generally unmethylated in normal cells [120]. Tissue-specific DNA methylation, particularly the genes’ promoter hypermethylation, is a valuable biomarker of early detection, prognosis, risk assessment, and disease recurrence [121,122,123]. One of the first analyses of the CpG island methylation pattern in 98 primary human cancers, using restriction landmark genomic scanning (RLGS), analyzed 45,000 unselected CpG islands. RLGS analysis demonstrated that an average of 600 CpG sites were methylated aberrantly in different tumors; many changes occurred in early-stage cancer cells [124]. Furthermore, the CpG-island methylation pattern was also observed among distinct tumor types. In the genome-wide search for methylated CpG islands in LCs, the microarray analysis of the methylated immunoprecipitated DNA (MeDIP-methylated DNA immunoprecipitation) has been performed on more than 50 k CpG islands (24 659 gene promoters described in Human Refseq and 28 226 islands annotated on the UCSC genome browser). MeDIP revealed 2414 genomic positions differentially methylated between the tumor and adjacent lung tissue, with the vast majority of CpG hypermethylated in LC that were annotated to 477 TSGs [125]. Some of the indicated genes, involved in the regulation of gene transcription, apoptosis, DNA binding, or cell adhesion, were significantly hypermethylated in cancer: *DMRTA2*, *EVX1*, *GATA3*, *HOXA2*, *HOXA10*, *IRX2*, *PCDHA12*, *POU3F4*, *PRDM14*, *SFMBT2*, *SHOX2*, and *TAL1* [125]. The silencing of *HOXA2* and *HOXA10* expression genes encoding the transcription factors was associated with worse prognosis [125].

Several differentially methylated genes were identified with the whole-genome DNA methylation profile analysis of the microarray data covering nearly half of the million cytosine positions in the human genome [126]. In addition, 3% of the analyzed CpG were differentially methylated in the lung AC; 60% of sites were hypermethylated (3492 genes), and 40% were hypomethylated (5860 genes). Among the silenced genes are the transforming growth factor ß (*TGFß*), Insulin-like Growth Factor 2 (*IGF2*), and HOX transcription factors family. Interestingly, the interleukins and chemokine ligand (c–C motif 3, 4, 8, 11 and 13, 14, 15, 26) genes having the immunomodulating activity were hypomethylated [126].

## 5. Role of Lung Inflammation in COPD Development and Lung Cancer

The progression of COPD is characterized by intense lung inflammation resulting from long-term tissue damage and acute inflammation induced by noxious particles [127]. COPD’s chronic inflammatory process involves innate and adaptive immunity and presents heterogeneity [128]. It results in both emphysema with parenchymal involvement and chronic bronchitis, which predominantly affects the small airways [129]. A characteristic feature of COPD is the presence of acute exacerbations, which are typically associated with accelerated inflammation. Important causes of these exacerbations include bacterial and viral infections and environmental factors [130], which are strongly associated with more frequent hospitalization and mortality [131]. In the majority of smokers, COPD symptoms develop many years after the initiation of smoking and they are diagnosed over the age of ≥45 years [131] with higher risk to II, III, and IV COPD stages [132]. Interestingly, it has been observed that smoking causes the senescence of the alveolar epithelial cells in a dose- and time-dependent manner. The senescence-associated beta-galactosidase activity and the p21(CIP1/WAF1/Sdi1) protein expression increase in the epithelial cells. Subsequently, epithelial cells secrete significant amounts of pro-inflammatory cytokines [133].

Due to the accumulation of inflammatory mucous exudates in the lumen and an increase in the tissue volume of the bronchial wall, the inflammatory process in COPD is persistent despite smoking cessation and progresses over time [134]. The increase in the tissue volume of the bronchial wall was characterized by infiltration of the wall by both innate (macrophages/neutrophils) and adaptive inflammatory immune cells (CD4, CD8, and B lymphocytes) that formed lymphoid follicles [135].

The factors that drive inflammation in COPD after smoking cessation have not been established. However, autoimmunity, embedded particles/heavy metals from smoking, and chronic bacterial infection have all been proposed to be involved [134]. In the study, autoimmunity was characterized by the presence of anti-elastin antibodies and T-helper type 1 [T(H)1] responses, which correlated with emphysema severity [136]. Next, the exacerbations in COPD patients were directly connected with high levels of cadmium and manganese in the lungs of patients with advanced COPD and infection with bacteria like *Haemophilus influenzae* (NTHi). In addition, it was shown that NTHi could activate lung T cells and cause the expression of reactive oxygen species and proteases in patients with COPD [137].

The inflammatory process in the pulmonary tissue of COPD patients is characterized by an influx of monocytes, neutrophils, CD8+ lymphocytes, and sometimes eosinophils [20]. The accumulation of macrophages in the alveoli, bronchioli, and small airways significantly correlates with the development of emphysema [138].

Biopsy analysis of COPD samples detected an increase in neutrophils and CD8+ cytotoxic lymphocytes in the mucosal epithelium and macrophages in the subepithelium [139]. Macrophages can be directly activated by cigarette smoke and are thought to play a critical role in sustaining the chronic inflammation in the pulmonary tissue of COPD patients [140]. Neutrophils can subsequently participate by responding to chemotactic factors released by macrophages, epithelial cells, and other resident cells. Activated neutrophils and macrophages can then contribute to developing tissue damage and emphysema by releasing ROS and proteinases [141,142].

As chronic inflammation is an essential process in COPD, pro-inflammatory mediators such as chemokines (e.g., interleukin (IL)-8) and cytokines (e.g., TNF-α) possibly play an essential role in the pathogenesis of COPD. These modulators of immune cell function are found in the sputum and bronchoalveolar lavage fluid of COPD patients [143]. Additionally, these cytokines were detected in the plasma/serum of COPD, which can point to the systematic burden effect of prolonged COPD inflammation [144].

## 6. Biological Mechanisms of COPD and Lung Cancer Development

### 6.1. Smoking in COPD and Lung Cancers

Smoking is the most common cause of COPD and LC, associated with approximately 85–90% of cases of exposure to tobacco smoke [145]. Since tobacco smoke contains several dozen chemical compounds with proven irritating and carcinogenic importance, the patient’s health is negatively affected by active smoking and passive smoking [146]. The number of cigarettes smoked daily (smoking intensity) and initiation of smoking at a young age increase the risk of developing lung cancer. With the duration of exposure to tobacco smoke (the period of addiction), the risk of developing LC increases significantly, but the risk is two to three times higher in men than in women [145].

Exposure to secondhand smoke (SHS) also plays a crucial role in lung cancerogenesis. SDS is formed from the side-stream smoke emitted into the environment from smoldering cigarettes and other tobacco products between puffs and from the mainstream smoke exhaled by the smoker [147]. Many studies have shown strong and consistent associations between SHS and various diseases, with the most substantial evidence for developing lung cancers, ischaemic heart disease, and asthma (new cases). In the case of SHS’s effect on the health of children, there is strong evidence for low birth weight, sudden infant death syndrome (SIDS), childhood chronic respiratory symptoms, lower respiratory illness in young children, asthma (new cases and exacerbation), middle-ear effusion, and infection in young children [148]. It has been estimated that SHS is independently the third leading preventable cause of death, and therefore, a significant public health burden [149]. Globally, 80% of the world’s smokers live in low- and middle-income countries (IARC/WHO World Cancer Report 2020) [148].

Smoking cessation attempts may reduce the morbidity and mortality due to lung cancers; however, difficulty in quitting increases with increased nicotine dependence and the number of prior quit attempts [150]. In people who quit smoking, the risk of LC gradually decreases; after many years, it is about twice as high as in non-smokers [145].

In experiments with animal models, exposure to cigarette smoke led to tissue remodeling resembling the changes in COPD [151]. The activity of the irritating agents in cigarette smoke and coal and liquid fuel fumes may disequilibrate the oxidant–antioxidant balance [152]. These imbalances may lead to the leakage of electrons from the electron transport chain, causing oxidative stress locally in the lung tissue [153]. In many investigations, reactive oxygen species (ROS), oxidative imbalances and activity are indicated as a process triggering the development of the NSCLC and COPD [154,155].

With the co-existence with impaired antioxidant defense, all these issues lead to oxidative and carbonyl stress. Both of these states play a significant role in COPD pathobiology and may account for the development of substantial comorbidities of this disease. On the other hand, in both COPD and LC, the leukocytes and macrophages will release ROS in the ongoing inflammation process in bronchial branches, which secondarily contributes to oxidative–antioxidative imbalance [154,156].

### 6.2. Oxidative Stress

Cellular damage or loss of viability in the air sacs of the lungs is commonly observed in patients with COPD. Risk factors, such as smoking and air pollution, aggravate and accelerate COPD symptoms based on molecular mechanisms connected with oxidative stress and the recruitment of inflammatory cells or neutrophils to the lungs [157].

Oxidative stress is caused by over-accumulation of the ROS and reactive nitrogen species (RNS), which leads to detrimental effects such as DNA damage, cellular damage, and premature aging. It acts as a trigger for several diseases [158,159]. ROS includes ions like hydroxyl radical (·OH) and superoxide anion (O2·−), which play an essential role in defense against pathogens and intercellular signaling. These free radicals possess unpaired electrons, which allow the oxidative transfer of electrons to other molecules, damaging amino acids, fatty acids, or even nucleic acids. It can also lead to further production of ROS or its inactivation. The human organism has several protective antioxidative mechanisms and antioxidant sacrificial proteins, such as albumin, mucin, and glutathione, and some antioxidant enzymes like superoxide dismutase (SOD), catalase, and glutathione peroxidase (GPx), which neutralize the ROS and prevent damage [160,161]. However, upon long-term oxidative stress exposure, these defense mechanisms fail, leading to imbalances in oxidant–antioxidant balance. This disproportion may lead to the leakage of electrons from the electron transport chain, causing oxidative stress locally in the lung tissue [154].

In the COPD patient context, free radicals start damaging the lung cells due to the oxygen-rich environment and good blood pulmonary circulation, which is prone to free radical production [162]. For smokers and people exposed to high levels of air pollution (including air-borne pathogens and toxins), the exposure to ROS is relatively high, increasing oxidative stress.

Consequently, oxidative stress on the molecular level leads to alterations in protein structure and function and triggers inflammatory cell response. The ROS and RNS alter amino acid residues, thus negatively affecting the secondary and tertiary protein structures and their function. Similar changes were observed due to thiol or amine modifications caused by oxidative stress conditions [163,164].

Certain inflammatory factors such as TNF-α, IL-8, and leukotrienes (LTB4) in cells lead to an inflammatory response and release of free radicals from cells along with proteases and cytokines [165]. This phenomenon activates the neutrophils and their recruitment in the lungs, which results in emphysema and bronchitis during COPD [142]. Therefore, the damaged lung tissues lose elasticity in the smooth muscle, and the secretion of mucus blocks the airways [166].

### 6.3. Protease Involvement and Matrix Remodeling

#### Role of Proteases in COPD and Lung Cancer

Several enzymes, including proteases, play an essential role in COPD disease progression. The three most important families of proteases involved in COPD progression include (i) serine proteases (responsible for alveolar tissue destruction), (ii) MMPs (induce severity of COPD), and (iii) cysteine proteases (control of cellular apoptosis) [167]. The function of these proteases is controlled by endogenous inhibitors (known as anti-proteases), which prevent their over-secretion and over-activation, and thus do not allow for their uncontrolled harmful effects. However, in COPD, this protease–anti-protease stability is disrupted and affects lung damage [168]; protease-inhibitors imbalance is enhanced by a decrease in antiproteases such as α1-protease, neutrophil elastase, and leukocyte protease inhibitors and secretory proteinase inhibitors, which are inactivated by oxidants.

Serine proteases neutrophil elastases (NE) play an anti-microbial role (especially in Gram-negative bacteria) during neutrophil phagocytosis to digest the microbial outer membrane proteins in a ROS-dependent mechanism [169,170].

In physiological conditions, NE is predominantly expressed on the cellular membrane, while 4–5% of NE is released extracellularly [171]. In COPD cases, a high concentration of NE in the extracellular membrane results from neutrophil necrosis [172].

This NE dysregulation results in several detrimental effects in the lungs, including mucus obstruction of the airways (accompanied by upregulated mucin *MUC5AC* gene expression) and bronchial epithelial cells. This phenomenon is connected with intracellular signals, such as activation of ROS, NADPH quinone oxidoreductase 1 (*NQO1*), and *EGFR* higher expression [173,174,175]. The higher NE level leads to the release of TGFα on the cell surface and increases the epithelial permeability by degradation of the junctional proteins (Zona occludins-1 and E-cadherin), which also activates the apical receptor, EGFR [176,177,178]. All these events increase goblet cell metaplasia and enhance mucus production in the airway epithelium [179]. High NE inflicts damage to ciliary structures, reduces their motility, and disturbs the hydration of the airway surface [180,181]. NE plays a significant role in COPD progression by degrading extracellular matrix (ECM) proteins such as elastin on the release of TGF-β1, thereby damaging the alveolar structure, causing emphysema and subepithelial fibrosis [182,183,184]. NE has also been found to activate several pro-inflammatory factors, such as IL-8 (by activating TLR4 and EGFR signaling pathways), leading to increased COPD severity in patients [166].

Other serine proteases involved in COPD progression include cathepsin G and proteinase 3, which are involved in degrading proteins such as elastin and phospholipid transfer protein) [185]. A study identified higher amounts of elastin neo-epitopes from cysteine G and proteinase 3 activity in COPD patients compared to normal individuals [186].

The MMPs are responsible for the degradation of the ECM components and are considered vital in tissue remodeling [187]. The elevated levels of these proteases have been implicated in lung damage and COPD development [188]. The MMPs most crucial for COPD severity include MMP-2, MMP-9, MMP-12, and MMP-13. Studies have indicated that MMP-12 interacts with the enzyme macrophage elastase in smokers, resulting in emphysema in COPD patients [189]. MMP-12 levels and activity from COPD patients’ sputum samples were directly related to the extent of emphysema measured from lung function [190]. Some genetic connections have also been identified linking *MMP-12* and its role in COPD progression. Individuals with homozygous A/A allele SNP in the rs652438 region in *MMP-12* have high chances of severe COPD [191]. These SNP have also been associated with emphysema and increased macrophage infiltration [192].

MMP-9 can degrade proteins such as fibronectin and elastin useful in the cellular invasion of the basal membrane by mononuclear phagocytes and synovial fibroblasts where it is present [193]. MMP-9 has been found to play a significant role in the migration of eosinophils, and its accelerated activation with MMP-2 was linked with airway inflammation in COPD [194,195]. Upregulation of MMP-9 was found in serum, sputum, and peripheral blood samples collected from COPD cases compared with non-COPD controls [14,196]. Additionally, the increased MMP-9 level was associated by the production of cough and decreasing FEV_1_ in a population-based cross-sectional study [197]. It plays a significant role in fibrosis during COPD due to its matrix remodeling properties [198,199]. Next, MMP-13 has also been found to be overexpressed in the lung tissues of COPD patients [200]. In a viral exacerbation model of COPD, MMP-13 was identified as responsible for lung structure destruction [200].

Cysteine proteases like caspase-3, caspases-8, and caspase-9 are essential in controlling apoptosis in COPD patients. High mRNA expressions of caspases were observed in COPD patients, where an increased number of alveolar epithelial and endothelial cells were found to be apoptotic [201]. In the case of the bronchial epithelial cells, apoptosis was found to be mainly due to caspase-8 activity, which was triggered by increased levels of the protein p53-a cell cycle regulator [201]. Next, cathepsin S was found at a high concentration level in the serum of COPD patients [202], and cathepsin type K was detected at a high level in lung homogenates from COPD patients exposed to chronic smoking [203].

Aspartyl protease–cathepsin E was observed to mediate the expression of the mitochondrial fission protein, dynamin-related protein 1, which destroys the lung parenchyma via caspase-dependent apoptosis in murine models [204]. It is directly related to airflow limitation and inversely to lung function in COPD patients [205].

Protease–antiprotease imbalance is one of the most crucial factors for COPD disease progression. The most critical antiprotease deficiency triggering COPD is that of α-1-antitrypsin (AAT), which plays a vital role in neutrophil elastase inhibition and other proteases such as proteinase-3, cathepsin G, caspase-3, and neutrophils serine protease-4 [206,207,208]. It was found that genetic mutations (a single allele of the Z α-1 antitrypsin) are responsible for AAT deficiency [209], and this defect results in a lack of NE regulation resulting in parenchymal destruction of the lung [210]. Other protease inhibitors, such as chelonianin, elafin, and secretory leukocyte protease inhibitors (SLPI) can inhibit NE [211]. Consequently, the deficiency of these proteases leads to emphysema, alveolar tissue damage, cellular apoptosis, and higher severity of COPD. 

Among potential genes of interest are those involved in ECM reorganization, which plays a crucial role in cancer development and progression. ECM remodeling accompanies many physiological processes like embryogenesis, angiogenesis, apoptosis, wound healing, and damage repair [212,213]. Physiologically, one of its primary functions is the degradation of collagen type IV, a basement membrane component [214].

In pulmonary carcinoma, the presence of MMP2 protein or elevated gene expression was demonstrated both in highly invasive areas and those of moderate growth (lepidic growth) [215], as well as in stromal fibroblasts [216]. It was also observed in preneoplastic bronchial squamous lesions [217]. The upregulation of *MMP2* metalloproteinase expression in NSCLC patients has been found to correlate with larger tumor size, lymph node involvement, and distant metastasis [218]. In ECM remodeling, the action of MMPs is countered by the endogenous tissue inhibitors of the metalloproteinases (TIMPs) family, i.e., these act as inhibitors of tumor growth and angiogenesis. One TIMP family member, the TIMP3 protein, inhibits tumor growth by preventing the adhesion of cells to ECM and promoting the activation of apoptosis via the caspase-8 pathway [219]. The TIMP3 protein explicitly inhibits the action of MMPs by non-covalently binding to them and protects the proteolysis of the ECM. Due to its pro-apoptotic function, TIMP3 is regarded as a tumor suppressor gene (TSG) [220]. *TIMP3* expression has been altered in many human cancers, i.e., gastric, hepatic, prostate, endometrial, and lung [221]. In NSCLC, *TIMP3* silencing by EZH2 protein, known to cause histone lysine methylation (H3K27), was linked to tumor progression and metastasis [215]. TIMP3 expression decreases with pTNM grade and negatively correlates with cancer staging and prognosis [222]. Downregulation of the *TIMP3* gene has been observed both in cancer tissue and in neighboring non-cancerous tissue and correlated negatively with miR-20a, which can point out the epigenetic silencing of the genes controlling the ECM remodeling. *TIMP3* downregulation observed in long-term smokers may be one of the examples of epigenetic protease deregulation and one of the molecular causes of cancerogenesis among SCC patients [223].

## 7. Diagnosis and Treatment of COPD

### 7.1. Detection of COPD

At present, little is known about when the earliest changes of COPD begin in susceptible individuals. Several authors suggest that these changes may start as early as in utero, progressing during childhood, for example, with recurrent infections, exposure to passive smoke, etc., and go on into adolescence, with further active and passive exposure to cigarette smoke, resulting in a reduction in the peak attained lung function, which subsequently increases the risk of being diagnosed with COPD in later life.

There is a great deal of uncertainty regarding the moment of COPD onset. The condition may develop in utero [224,225], stay dormant or progress during childhood, and acquire the detectable phenotype only in adulthood. Next, external risk factors, such as active and passive exposure to cigarette smoke can significantly accelerate disease development at any age. The importance of early COPD diagnostics is emphasized by all research that explicitly stresses the difference between early and mild forms of COPD [226]. A mild form of COPD describes the degree of airflow obstruction, which can be persistent for several years, while early COPD describes the time point and substantial processes of the disease onset. Unfortunately, these two terms were used interchangeably at the dawn of COPD research, which distorted present statistical data. The situation is worsened by the uncertainty in the early COPD stage definition due to the heterogenicity of the disease.

Traditionally, COPD is diagnosed by the method of spirometry, which detects FEV_1_/forced vital capacity (FVC) ratio. The post-bronchodilator administration ratio FEV_1_/FVC < 0.70 indicates the presence of persistent airflow obstruction and confirms the presence of COPD. To date, spirometry is the only instrumental method used for the clinical diagnostics of COPD, as it is considered the most reproducible and objective for measuring airflow obstruction. Nevertheless, accumulating evidence demonstrates that the exclusive use of spirometry as the only diagnostical approach is not enough. SPIROMICS study [227] showed that patients with preserved pulmonary function, meaning no present evidence of airflow abruption, may have other respiratory symptoms, such as reduced exercise capacity and more frequent exacerbations compared with asymptomatic patients, indicating the spirometry method to be sufficient for the diagnosis of the established disease but not its onset. In addition, patients with the same numerical degree of airflow obstruction can obtain different severity of other COPD symptoms and a different course of the disease [228].

### 7.2. COPD Patient Classification and Treatment

**Figure 2:**
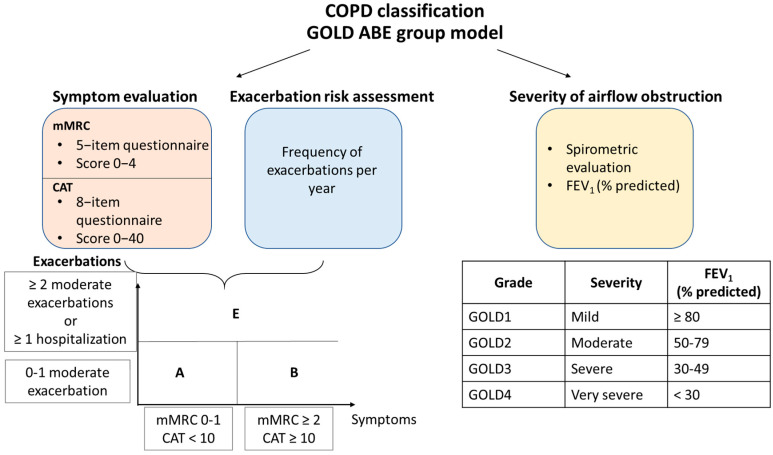
COPD assessment. GOLD ABE group model. Abbreviations: mMRC, the modified Medical Research Council dyspnea scale; CAT, the COPD Assessment Test; FEV_1_, forced expiratory volume in 1 s.

In 2022, the Global Initiative for Obstructive Lung Disease (GOLD) proposed a combination of two evaluation systems for COPD patient classification (https://goldcopd.org/archived-reports/, accessed on 15 December 2022). The first one presents a spirometry scoring system, i.e., grading the patient from one to four based on the percentage prediction of FEV_1_, and the second shows the separation of patients into four groups (A, B, C, or D) based on the symptom severity and the exacerbation occurrence rate in the past year. Recently, the new system was suggested in the 2023 Gold Report (https://goldcopd.org/2023-gold-report-2/, accessed on 15 December 2022), where groups C and D are combined into a group E, highlighting the clinical relevance of exacerbations (Figure 2). Thus, the modern assessment system considers structural anatomical changes, resulting in the decrease of predicted percentage FEV_1_, symptomatic pattern, and the dynamic of exacerbation, thereby reflecting the change towards personalized analysis and choice of individual COPD treatment.

Circulating cancer cells (CTCs) in peripheral blood have already shown diagnostic and prognostic utility in oncology [229]. In studies with computed tomography scan, CTCs were detected in patients with COPD (without clinically detectable LC). This strongly highlighted the value of this parameter for monitoring CTC-positive COPD patients for early LC diagnosis [230]. Furthermore, it was shown that alveolar cells may be discharged into the bloodstream and that these cells could be identified in the peripheral blood of patients with COPD with liquid biopsy techniques. In the studies, circulating pulmonary cells or remnants of these cells were detected in the peripheral blood of COPD patients (using immunoreaction against hyaluronan receptor CD44v6-specific marker of lung tissue) and showed significant correlations [231].

Due to the lack of knowledge about the origins of the disease, currently there is no cure to halt the progression of COPD. Existing therapy is aimed solely at relieving the symptoms, slowing down the progression of the disease, reducing the frequency of disease exacerbations, and improving the quality of life. The spirometric FEV_1_ parameter was shown to be essential for COPD diagnostics, mortality prediction, and hospitalization at the population level, but more is needed for the estimation of individual patients and choice of therapy. Currently, belonging to a particular group of the GOLD ABE model is the critical parameter for evaluating the patient’s condition and treatment choice. Pharmacological treatment can be divided into initial and follow-up therapy. Initial treatment is determined based on the ABE group of the patient and consists mainly of short- or long-acting bronchodilators. Follow-up is based on the clinical response to the previous medication and the development of side effects like pneumonia, review dyspnea, and exacerbation risks (Table 3). In the treatment of COPD, much attention is paid to smoking cessation, which is vital for all patient groups [232], and to the monitoring of bacterial and viral infections, which may lead to rapid COPD progression and exacerbations, so vaccination is highly recommended [233].

Although COPD predisposes to LC, these conditions are different and heterogeneous in their pathogenesis and require other treatments. Meanwhile, the chosen treatment methods for one disease should not complicate the course of another. In some instances, COPD treatment cannot give a beneficial outcome in reducing the incidence of cancer. Thus, antioxidant therapy with N-acetyl cysteine (NAC) for COPD was shown to provide inconsistent results in preventing cancer development [234]. A nationwide cohort study in Taiwan showed that patients with COPD treated with one, two, or more medications had a 2.6-fold, 3.0-fold, and 3.3-fold risk of any cancer development, respectively [235]. In addition, in the case of joint diseases, the choice of LC therapy should consider the state of COPD, such as impaired FEV_1_ percentage, emphysema, etc., and be safe for COPD patients’ treatment [236]. In this way, treatment of one disease should be specific and based on the therapy history of another illness, and consider possible side effects potentially influencing their progression.

In our understanding, current difficulties in COPD diagnosis and treatment emerge due to a lack of understanding of the etiology of this disease in each patient. Considering the current problem of COPD classification and the possible overlap of its subtypes in one clinical case, we can assume a variety of starting points for the development of the disease and the complexity of its evolution, which need to be carefully studied. State-of-the-art research methods, such as single-cell sequencing, multi-omics [237], the development of COPD models [238], and inflammatory microbiome studies [239], may shed light on the molecular mechanisms of COPD development.

## 8. Targeted Therapy for Lung Cancer

Intensive research in the pharmacological treatment of LC highlights the promising results in the direction of molecularly and immunologically targeted drugs [112]. Advanced molecular diagnostics is possible thanks to the latest molecular techniques enabling the identification of specific mutations and rearrangements of genes, which are predictive and allow for individualization of therapy in many cases. The tremendous success in the systemic treatment of LC is the introduction to the clinical practice of personalization of the procedure and the sequential application of subsequent—often different in terms of the mechanism of action—treatment methods. Modern treatments, such as molecular targeting drugs and immune checkpoint inhibitors, are increasingly combined with standard therapies [240].

Many inhibitors have already been proposed to target tyrosine kinases-epidermal growth factor receptor (TKI-EGFR), inhibitors of ALK, ROS1, BRAF, NTRK, and immunotherapy using monoclonal antibodies, mainly against the programmed cell death one receptor (PD-1, programmed cell death 1) or its ligand (PD-L1, programmed cell death ligand 1) to improve the therapeutic efficacy of NSCLC [241]. PD-1 is expressed by T-cells, and PD-L1 expression by the tumor can suppress the T-cell–mediated cell death. PD-1/PD-L1-blocking drugs inhibit the adverse regulatory effects of the PD-1/PD-L1 pathways, resulting in the restoration of the initial anti-tumor immune response [242].

Among the tyrosine kinases inhibitors, the broad selection of FDA-approved drugs targets the activating mutation in the EGRF. Presence of EGFR mutations is more prevalent among young women, non-smokers, and patients of Southeast Asian ethnicity (mainly China and Japan) [243]. EGFR targeting TKIs, erlotinib and gefitinib (first-generation), and osimertinib (second-generation), established longer progression-free survival (PFS) in EGRF-positive patients (with locally advanced or metastatic cancers), versus platinum-based doublet chemotherapy [244,245]. The anti-EGFR treatment also comprises monoclonal antibodies (mAbs) such as necitumumab, blocking the EGFR ligand binding site and downstream processes [246].

Third-generation drugs could counter resistance against first-generation TKI-EGFR, e.g., osimertinib can act against resistant mutations like T790M [247]. KRAS inhibitors such as the G12C mutant protein have been found to be very effective against severe TKI-EGFR resistance. Similarly, therapy options based on HER-3 targeting can be used to counter resistance against TKI-EGFR. Compounds such as U3-1402 and patritumab (U3-1287) combined with a TKI-EGFR, erlotinib, can act against EGFR-resistant NSCLC cells [248]. Several miRNAs have been found to affect drug resistance by affecting the PI3K/AKT/mTOR pathway. Targeting miRNAs such as *miRNA-328*, *miR-21*, and *miR-23a* showed a reversal of resistance against cisplatin, gefitinib, and erlotinib, respectively [249]. Attempts have also been made to combine the *miR-34a* with siKRAS and cisplatin to increase the efficacy of the drug against the KRAS/P53 mutation [250]. JAK-2 inhibitors such as ruxolitinib and CYT387 (in combination with cetuximab) have shown good efficacy against resistant NSCLC cell lines [251]. On the other hand, treatment with tyrosine kinase inhibitors is more effective than standard chemotherapy, which can be seen by improving the quality of life of patients, and also shows a different toxicity profile.

According to the NCCN Guidelines for NSCLC, it is recommended to test NSCLC patients for *ALK* and *ROS1* rearrangements. In up to 5% of NSCLC patients, particularly those with AC subtype and non-smokers, the *EMLK4*-*ALK* rearrangements are detected [108]. One of the drugs targeting the anaplastic lymphoma kinase rearrangements is alectinib, an oral TKI recommended as a preferred first-line treatment for metastatic patients [252,253]. Other ALK-targeted TKIs suggested are crizotinib and ceritinib. In case of progression, the secondary treatment can include another TKI (e.g., alectinib, ceritinib, after first line crizotinib) or cytotoxic chemotherapy (after first line alectinib or ceritinib) [108]. Crizotinib is also approved as a first-line therapy for patients with the *ROS1* fusion protein (ROS1 has high similarity to ALK) having locally advanced or metastatic NSCLC [254]. Crizotinib has a very high response rate of 70–80% [255].

Therefore, it is essential to perform molecular determinations in tissue/cytological material in patients with NSCLC before qualification for systemic chemotherapy, and in the case of finding molecular disorders, to apply molecularly targeted therapy in the first-line treatment [256].

## 9. COPD and Lung Cancer Connection

**Figure 3:**
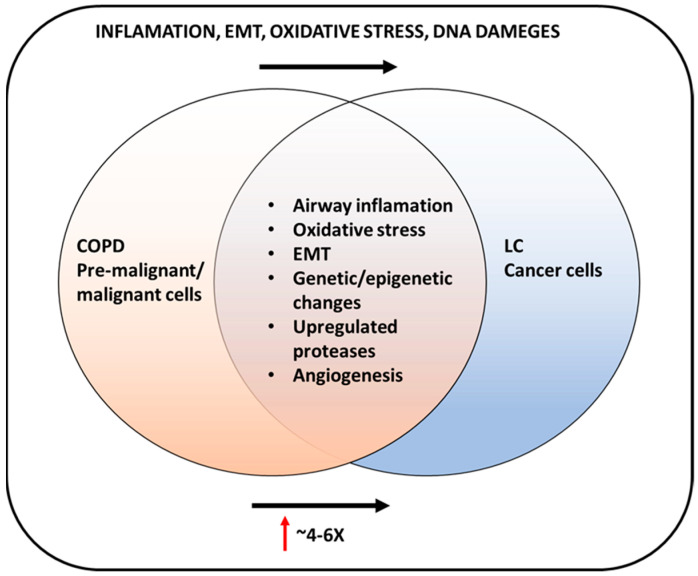
Increased lung cancer in chronic obstructive pulmonary disease (COPD). Inflammation and increased oxidative stress in COPD may enhance the growth and metastasis of lung cancer.

COPD is an independent risk factor for lung carcinoma, particularly squamous cell carcinoma, and LC is up to six times more likely to occur in smokers with airflow obstruction than in those with normal lung function [6,257] (Figure 3). The meta-analysis of the associations of the previous lung diseases as risk factors for lung cancer, performed by Brenner et al., revealed that COPD, emphysema, chronic bronchitis, pneumonia, and tuberculosis conferred relative risks of LC formation firmly in the smoking population [258]. The increased risk of LC development in the case of never-smoking patients may be explained by the inflammatory response induced by the primary disease within lung tissue. Previous lung conditions are known to cause an inflammatory response in the lung [154,238,258]

The retrospective study by Wang et al. comparing the COPD-NSCLC versus non-COPD NSCLC patients showed that COPD-NSCLC patients developed the squamous cell carcinoma histological subtype more often. It was demonstrated that the overall survival time (OS) was significantly shorter for LC cases with COPD comorbidity [259]. The neutrophil to lymphocyte ratio (NLR), related to inflammation, was elevated in the NSCLC-COPD group but was also considered a predictor for shorter OS. The elevated NRL was proposed as an independent inflammatory marker for COPD aggravation [260]. The increased NLR (pretreatment NLR level ≥5) was also correlated with worse outcomes in NSCLC patients treated with nivolumab [261]. Elevation of the neutrophils vs. lymphocytes is associated with deterioration of the immune system response, observed in chronic inflammation.

The high prevalence of lung cancer in COPD suggests that there may be common mechanisms, such as premature aging in the lungs, genetic predispositions to either disease, or common pathogenic factors (such as growth factors, activation of intracellular pathways, or epigenetics).

GWAS studies in significant COPD and LC cohorts have found the same risk *loci*, including *CHRNA3* and *CHRNA5* SNPs (15q) and regions at 4q31 (*HHIP*), 4q24 (*FAM13A*) and 5q (*HTR4*). Nicotine smoking may explain the overlap in risk loci between LC, smoking behavior, and COPD. In addition, EMT and inflammation are pathogenic features of COPD and lung cancer [262]. The global methylation pattern analysis, using an epigenome-wide association study (EWAS), revealed that downregulation of the *CCDC37* and *MAP1B* genes was associated with COPD and LC [263]. Examination of the epigenetic regulation of human mitochondrial mtTFA, associated by its function with oxidative stress and inflammation, revealed groups of silenced (*AIMP1*, *IFNG*, *LTA*, *LTB*, *TNF*) and activated genes (*BMP2*, *CCL2*, *IL5*, *VEGFA*) for both LC and COPD [264].

Another recent experiment identified a correlation between COPD-associated genes that can cause SCC using a genetic network analysis software named Cytoscape. They identified the differential expression of genes associated with heat shock proteins (90 α family class A member 1 (*HSP90AA1*) and family B (small) member 1 (*HSPB1*)), adrenoceptor β2 (*ADRB2*), and transducin β like 1 X-linked receptor 1 (*TBL1XR1*), for both SCC and COPD [265].

Several microRNAs were identified to be upregulated both in COPD and lung cancer. For example, miR-1 has been linked to cigarette smoking-related conditions, including cancer, and is downregulated in the skeletal muscle of patients with COPD compared with non-smoking controls and expression correlated with clinical features. The activity of *miR-1*, *miR-21*, and *mir-146a* has been altered in the inflammation process and cancer proliferation. NF-κB (nuclear factor κB) activating the *miR-146a* was implicated as a causal link between inflammation and carcinogenesis [266]. Fathinavid et al. [267], through a pathway enrichment analysis method, identified the miRNAs implicated in common pathogenesis mechanisms between COPD and NSCLC. Among three common pathways: (1) non-small cell lung cancer, (2) cell cycle, and (3) p53 signaling pathway, the following miRNAs were upregulated *miR-15b*, *miR-106a*, *miR-17*, *miR-103*, and *miR-107. MiR-107* targets the epidermal growth factor receptor (EGFR) [267]. *EGFR* overexpression has been observed in NSCLC and COPD and is inversely correlated with %FEV1.0 [268].

## 10. Conclusions

COPD not only contributes to the development of LC, but the significant overlap between COPD and LC symptoms might lead to a delay in recognizing an LC diagnosis [269].

LC with COPD comorbidity patients more commonly suffer from sputum retention, respiratory failure, and air leak. Patients operated on due to lung cancer, also suffering from COPD, are more vulnerable to post-operative pulmonary complications and prolonged hospital stay [270].

The many genetic, epigenetic, and biological factors involved in COPD and LC development are closely related.

Early detection of COPD and LC is urgently needed, so further studies investigating the links between critical biomarkers can elucidate the molecular signals responsible for COPD and LC development. More studies can provide insights into the creation of novel diagnostic and prognostic tools for early intervention and personalized therapeutic strategies.

Clarifying the molecular mechanisms of COPD and LC can improve early diagnosis and more efficient treatments.

## Figures and Tables

**Table 3:** GOLD group classification and treatment of COPD. Abbreviations: SABA, short-acting beta-agonists; SAMA, short-acting antimuscarinic; LABA, long-acting beta-agonist; LAMA, long-acting antimuscarinic; ICS, inhaled corticosteroids.

		A	B		E
Pharmacological therapy	Initial therapy	SABA or SAMA or LABA or LAMA	LABA or LAMA or LABA + LAMA		LAMA or LABA + LAMA or LABA + LAMA + ICS
	Follow-up therapy	Persistent dyspnea: (1) LAMA or LABA monotherapy → LAMA + LABA (2) LABA + LAMA → LAMA + LABA + ICS (3) Investigation and treatment of comorbid conditions			
		Exacerbations: (1) LABA or LAMA → LABA + LAMA or LABA + LAMA + ICS (2) LABA + LAMA → LABA + LAMA + ICS or roflumilast or azithromycin (3) LABA + LAMA + ICS → roflumilast or macrolide or LABA + LAMA			
Non-pharmacological therapy	Essential	Smoking cessation		Smoking cessation Pulmonary rehabilitation	
	Recommended	Physical activity			
	Optional	Flu vaccination Pneumococcal vaccination Pertussis vaccination COVID-19 vaccination			

## Data Availability

Not applicable.
